# Computational Analysis of Cardiovascular Hemodynamics

**DOI:** 10.1155/2012/260396

**Published:** 2012-09-06

**Authors:** Eun Bo Shim, Thomas Heldt, Akira Amano, Kyehan Rhee

**Affiliations:** ^1^Kangwon National University, Chuncheon-si 200-701, Republic of Korea; ^2^Massachusetts Institute of Technology, Cambridge, MA 02139, USA; ^3^Ritsumeikan University, Kyoto 603-8577, Japan; ^4^Myongji University, Yongin-si 449-728, Republic of Korea

## 1. Introduction

The human body requires a complex circulatory system to supply nutrients to, and to remove metabolic waste products from, its tissues. Given this primary purpose, circulatory function is closely related to the hemodynamic characteristics of blood vessels. This includes not only macroscale fluid dynamics, but also mass transfer in the microvasculature. Many experimental and clinical studies have examined these characteristics of vascular function. Over the past 50 years, mathematical modeling has become a powerful adjunct to such studies, as modeling provides a rational framework within which to analyze the cardiovascular system. 

Many mathematical models of the cardiovascular system have been developed since Grodins [1] published the first system-level dynamic cardiovascular model in 1959. Today, models of cardiovascular function exist on nearly every biological time and length scale. The design of such models naturally depends on the purpose of the underlying scientific questions, and the methodologies employed vary accordingly. Some subjects that have been extensively studied include hemodynamic models of specific vascular beds, such as the coronary or cerebral circulation [2]; the distributed impedance of the arterial and pulmonary trees [3]; lumped models of the integrated cardiovascular system [4]; detailed models of the fluid-structure interaction in specific vascular beds [5]. This special issue focuses on physiological and computational issues as they relate to the development of vascular models.

## 2. Brief Introduction of the Papers

Hemodynamic modeling draws upon the interdisciplinary field of vascular physiology, system engineering, fluid dynamics, and computer science. While the papers in this special issue reflect the broad constituency of this field, the main focus rests with the computational analysis of hemodynamic models.

The contribution by W. Jeong and K. Rhee reviews models dedicated to understanding the development, progression, and rupture of cerebral aneurysms. S. Nobari and coworkers utilize fluid-structure interaction models to study the effect of increased vascular stiffness on coronary blood flow. The contribution by J. Y. Park addresses the practical question of how to design a venous cannula for optimized flow. The study by P. Vasava and co-workers focuses on the variation in wall shear stress with hypo- and hypertension in a finite-element model of the aortic arch. W. Meng and coworkers model the effects of varying low-density lipoprotein (LDL) and high-density lipoprotein (HDL) concentrations on the wall shear stress of the carotid bifurcation. W. Kroon and coworkers present a novel scheme to couple one- and zero-dimensional wave propagation models. Finally, I. Chaichana, Z. Sun, and J. Jewkes analyze the effects of simulated plaques in a model of the left coronary artery.

## 3. Concluding Remark

Modeling studies like these provide an integrated framework to understanding vascular function. Research in computational modeling of the circulation will increase our understanding of normal vascular physiology and its pathophysiological aberrations in disease. The main motivation of the special issue was to highlight current and original research in computational modeling of cardiovascular hemodynamics and thus to encourage the applications of computational models of cardiovascular hemodynamics to clinical cases. We think the contributions in this issue achieve this goal.

*Eun Bo Shim* *Thomas Heldt* *Akira Amano* *Kyehan Rhee*
